# Mechanical evaluation of tibial fixation of the hamstring tendon in anterior cruciate ligament double-bundle reconstruction with and without interference screws

**DOI:** 10.6061/clinics/2020/e1123

**Published:** 2020-04-20

**Authors:** Anderson de Aquino Santos, Mario Carneiro-Filho, Roberto Freire da Mota e Albuquerque, João Paulo Freire Martins de Moura, Carlos Eduardo Franciozi, Marcus Vinícius Malheiros Luzo

**Affiliations:** I Departamento de Ortopedia e Traumatologia, Escola Paulista de Medicina, Universidade Federal de Sao Paulo, Sao Paulo, SP, BR; II Instituto de Ortopedia e Traumatologia (IOT), Hospital das Clinicas HCFMUSP, Faculdade de Medicina, Universidade de Sao Paulo, Sao Paulo, SP, BR

**Keywords:** Anterior Cruciate Ligament Reconstruction, Knee Lesions, Surgical Fixation Devices, Mechanics

## Abstract

**OBJECTIVE:**

The objective of this study was to compare two postero-lateral bundle (PLB) tibial fixation techniques for the reconstruction of the anterior cruciate ligament with double bundle: a technique without the use of an interference screw, preserving the native tibial insertion of the tendons of the gracilis and semitendineous muscles, and a technique with the use of an interference screw and without preserving the insertion of the tendons.

**METHODS:**

A comparative study was conducted in cadavers with a universal mechanical test machine. In total, 23 cadaver knees were randomized for tibial fixation of the PLB using the two techniques: Maintaining the tibial insertion of the tendons during reconstruction, without the use of an interference screw (group A, 11 cases); and fixating the graft with an interference screw, without maintaining the insertion of the tendons (group B, 12 cases). A continuous traction was performed (20 mm/min) in the same direction as the produced tunnel, and force (N), elongation (mm), rigidity (N/mm), and tension (N/mm^2^) were objectively determined in each group.

**RESULTS:**

Group A exhibited a maximum force (MF) of 315.4±124.7 N; maximum tension of 13.57±3.65 N/mm^2^; maximum elongation of 19.73±4.76 mm; force at the limit of proportionality (FLP) of 240.6±144.0 N; and an elongation at the limit of proportionality of 14.37±6.58 mm. Group B exhibited a MF of 195.7±71.8 N; maximum tension of 8.8±3.81 N/mm^2^; maximum elongation of 15.3±10.73 mm; FLP of 150.1±68.7 N; and an elongation at the limit of proportionality of 6.86±2.42 mm. When comparing the two groups, significant differences were observed in the variables of maximum force (*p*=0.016), maximum tension (*p*=0.019), maximum elongation (*p*=0.007), and elongation at the limit of proportionality (*p*=0.003).

**CONCLUSION:**

The use of the native insertion of the semitendineous and gracilis tendons, without an additional fixation device, presented mechanical superiority over their fixation with interference screws.

## INTRODUCTION

Approximately 250,000 lesions of the anterior cruciate ligament occur annually in the United States of America (USA) 1, and 60,000–175,000 ACL reconstructions are performed each year 2. An increase in the number of ACL reconstructions has been observed in recent decades in the USA, particularly in women below the age of 20 and in patients over the age of 40 3,4,5.

Currently we dispose of various surgical techniques that aim to restore articular stability; however, there is no consensus as to which type of reconstruction is more efficient 6-9. Single band reconstruction, which mainly reproduces the anteromedial band (AMB), has satisfactory results, yet approximately one fifth of patients may continue to have rotational instability 10-13.

With the intention of diminishing residual rotational instability, there has been an increase in interest in ACL reconstruction, with reproduction of both bundles of this ligament 10,14,15. With regards to ACL reconstruction with the double bundle, traditionally, it is necessary to make four bone tunnels; a femoral tunnel and a tibial tunnel for each bundle that constitutes the ACL. The great majority of these procedures use grafts from the tendons of the semitendineous and gracilis muscles. Usually the graft is fixed using a fixation device on the femur and another on the tibia for each bundle. The fixation of each bundle is performed in varying degrees of flexion, with the goal of achieving behavior of the neo-ligament as close as possible to that of the native ACL, enabling, from a biomechanical point of view, an improvement in the kinematics of the reconstructed knee 16.

**Figure 1:**
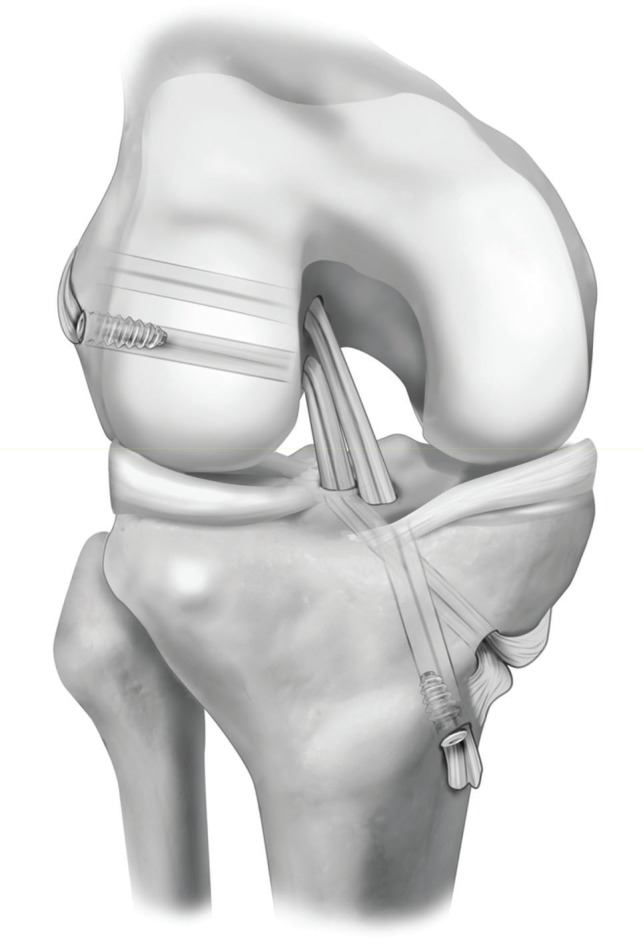
ACL reconstruction technique with double bundle described by Carneiro et al.

Currently, there are several fixation possibilities for the ACL graft, including metallic or bioabsorbable interference screws, cortical support fixations, and transverse fixations, although there is no consensus as to which is the best method 17,18. As described by Macey in 1939 19 and Cho in 1975 20, the fixation of the graft on the tibia may be performed to preserve the insertion of the tendons of the gracilis and semitendineous muscles, and without the requirement of additional fixation. Based on these citations, Carneiro et al. 21 developed a technique in which it is possible to obtain tension independently of each bundle, using only two interference screws, as opposed to the usual four used in double band reconstructions (Figure 1). A more economic and equally efficient way to perform the anatomic reconstruction of the ACL with a double bundle is possible if the insertion of the tendons that comprise the *pes anserinus* presents a resistance similar to that of the fixation via the interference screw.

The objective of this study was to perform a controlled mechanical evaluation of tibial fixation of the postero-lateral bundle (PLB) in ACL reconstruction maintaining the native tibial insertion of the tendons of the gracilis and semitendineous muscles without the use of an interference screw 21 *versus* tibial fixation of the PLB with the use of an interference screw, without preserving the insertion of the tendons.

## MATERIAL AND METHODS

The project was approved by the Ethics Committee in Research. The study was performed in cadaver knees that were acquired and stored appropriately. The specimens were preserved frozen at -20°C for up to 3 months and were unfrozen on the day preceding the trials. All the knees were used before they had been removed from the freezer in excess of 50 hours, with the aim to preserve the biomechanical properties of the bone, avoid jeopardizing the tests, and allow a greater similarity between *in vitro* and *in vivo* results 22.

**Figure 2:**
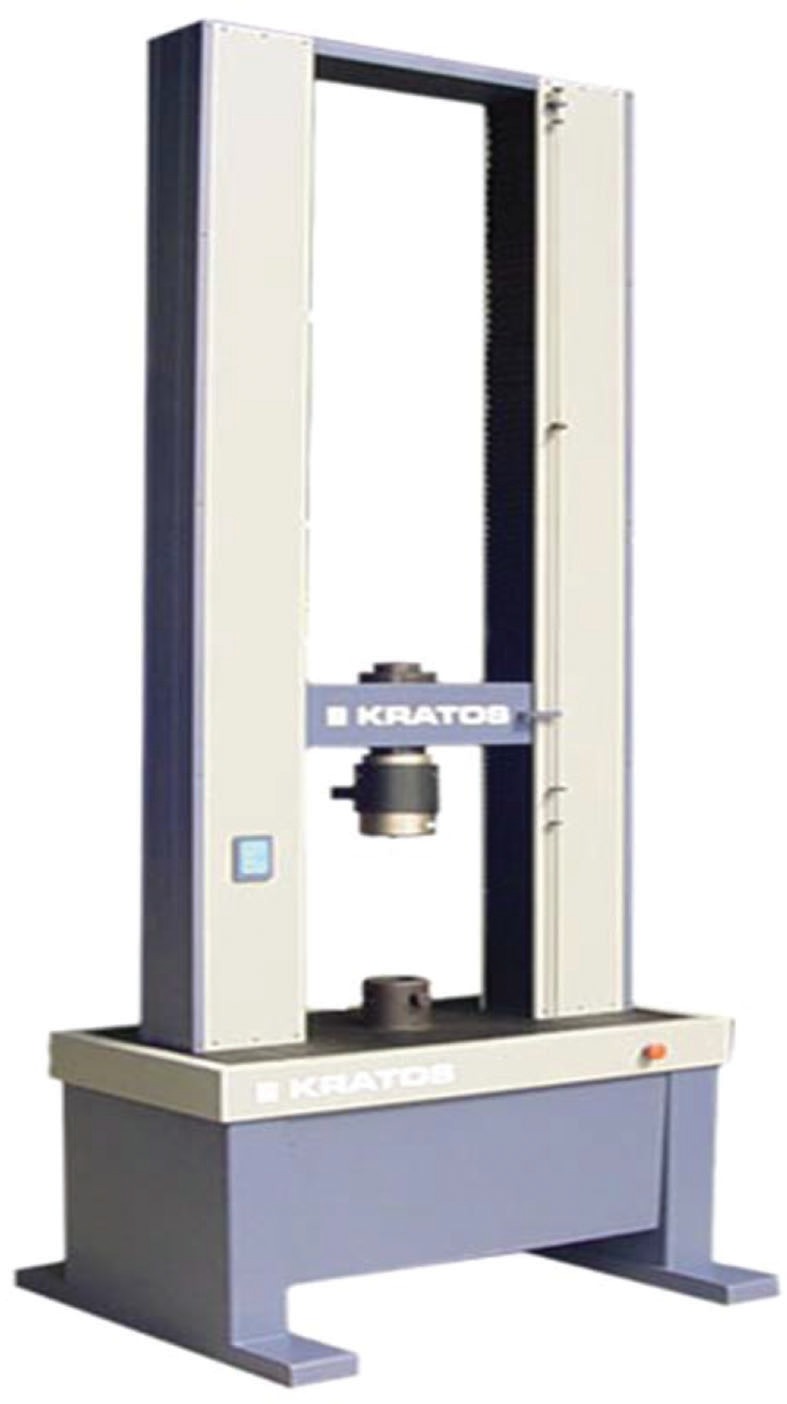
The Kratos universal mechanical testing machine.

The study was performed in conjunction with the Faculty of Medicine of the University of São Paulo, which provided the knee specimens through the Death Verification Service of the Clinical Hospital, and made available the Biomechanical Laboratory of the Institute of Orthopedics and Traumatology of the University of São Paulo, with a universal mechanical testing machine (Cotia, SP, Brazil; Kratos Equipamentos Industriais, model k5002); the machine had 100 kgf load cells and was connected to a computer with a system for acquiring the data necessary for a biomechanical study (Figure 2).

The adopted exclusion criteria were as follows: Insufficient flexor tendons, less than 20 cm in length individually; evidence of previous knee procedures and indicatives of an advanced osteoarthritic process, such as osteophytes; important misalignment and extensive erosion of the articular cartilage; and signs of systemic diseases on the knee joint, such as collagenosis and rheumatologic disease.

From an initial sample of 25 knees, 2 were excluded; 1 had flexor tendons that were insufficient in length, and the other exhibited signs of osteoarthritis with extensive osteophytes and important degeneration of the articular cartilage. Therefore, considering the exclusion criteria, 23 cadaver knees were randomized for the tibial fixation of the PLB by maintaining the tibial insertion of the tendons in the reconstruction, without using interference screws (group A, 11 knees) and by fixating the graft with interference screws (group B, 12 knees).

The mean age of the knees was 58.2±12.7 years, and there was no significant difference between group A (59.3±10.7 years) and B (57±13.1 years).

For the trial, we used only tibia with a length of 15 cm from the articular line. In all specimens, the tibial insertion of the native ACL was removed. To remove an autologous graft of flexor tendons from each cadaver, a vertical incision was performed on the anteromedial aspect of the proximal tibia, 3 to 4 cm from the articular interline, on top of the pes anserinus.

In group A, insertion of the the gracilis and semitendineous tendons was preserved. Through careful dissection, the proximal extremities were disinserted from the muscle, one at a time, with the aid of fenestrated tenotomy instruments. In group B, the tendons were disinserted from their tibial insertion and fixated with an interference screw in the tibial tunnel.

The thickness of the combined tendons, semitendineous, and gracilis was measured after rehydration with saline. Three sequential measurements were observed and the average thickness was obtained. The width of the graft was also measured, and the area of the graft was determined by considering the thickness measured multiplied by the width of the graft.

**Figure 3:**
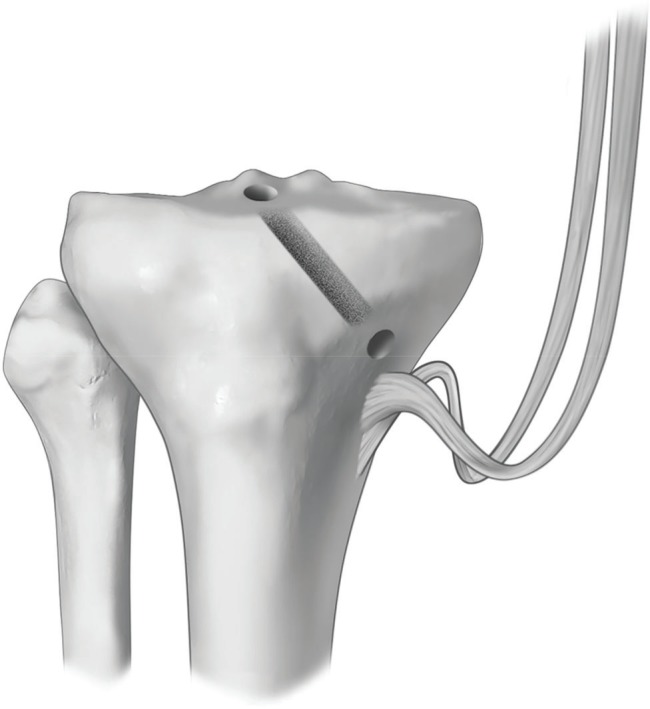
Illustration of the dissected hamstring tendons and their maintained insertions. PLT: Posterolateral tunnel (relative to the posterolateral bundle).

The size of the tibial tunnel was determined from the diameter of the double graft, which comprised one strand of the semitendineous and one strand of the gracilis; the measurement was obtained by passing the graft through a fenestrated meter with measures varying from 6 to 11 mm, with a 1 mm progression after each measurement. Thus, the tibial tunnel was created starting 0.5 cm proximal to the insertion of the pes anserinus and ending on the posterolateral area of the tibial insertion of the ACL. This tunnel was situated in a 45° plane in relation to the coronal plane. It was perforated using a tibial guide, guidewire, and drill with the same width as the double graft, from the outside in (Figure 3). In group B, in which the insertions of the hamstring were not maintained, the fixation of the graft was performed through the use of a metallic interference screw that was 1.0 mm larger than the diameter of the tunnel.

**Figure 4:**
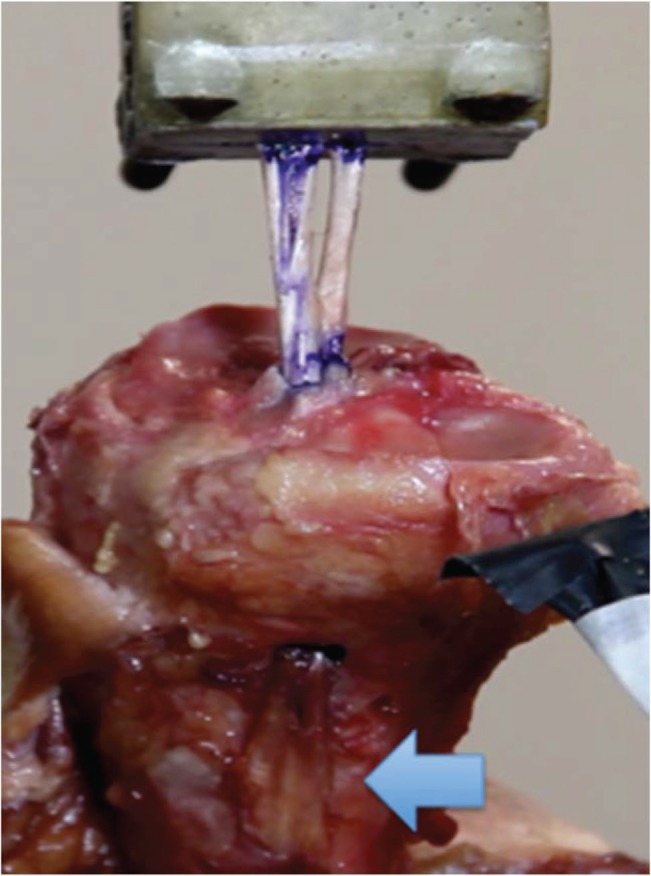
Photo of an anatomical piece of the tibia from group A (fixation without the screw), showing the mechanical trial with the Kratos apparatus in which the tibia was maintained on an immobile support. Arrow: Preservation of the tibial insertion of the hamstring tendons.

**Figure 5:**
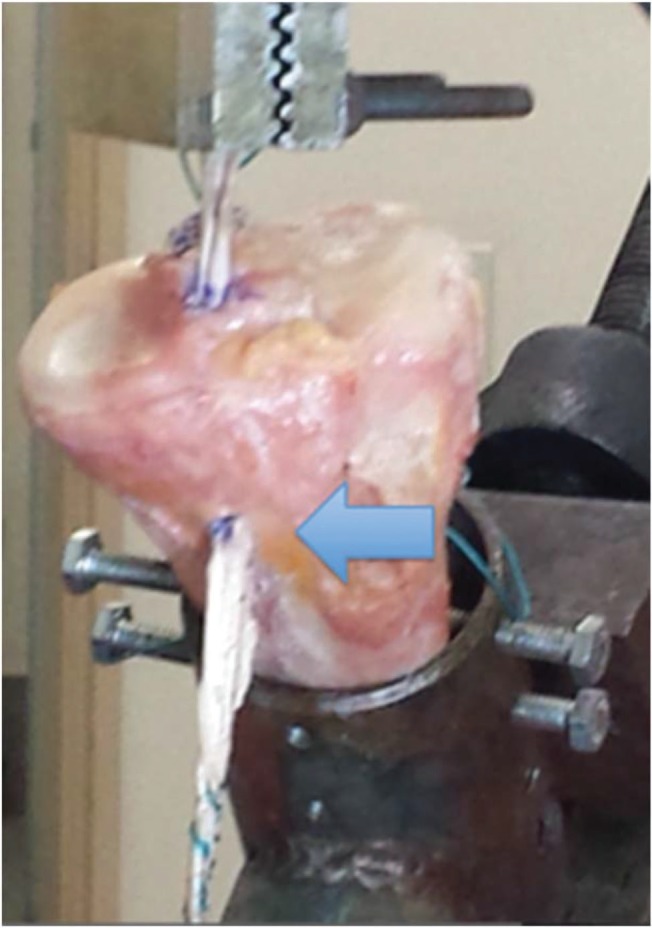
Photo of an anatomical piece of the tibia from group B (fixation with screw) showing the mechanical trial with the Kratus apparatus in which the tibia was maintained on an immobile support. Arrow: Freed insertion of the hamstring tendons fixed with an interference screw inside the tunnel.

With the distal portion of the tibia fixed on an immobile support, we proceeded to position the graft through the perforated tunnel. The free extremity of the graft, the distal 50 mm, was sutured with a non-absorbable Ethibond n°5.0 thread, and fixated, with the use of a hook, to the universal testing machine. Continuous traction was applied (20 mm/min) in the same direction as the produced tunnel, angling the piece as necessary and fixating it on the immobile support at the end. Force (N), elongation (mm), rigidity (N/mm), and tension (N/mm^2^) were objectively determined in groups A and B (Figures 4 and 5).

The maximum force at failure (MF) is represented graphically by the higher peak force reached. The force at the limit of proportionality (FLP), which can be graphically determined by the Johnson method, consists of a tangent point between the curve and a line with an inclination 50% smaller than the line that represents the linear region of the graph. If, at this point, the load were to be removed slowly, the piece would return to its original size 23. The FLP represents the beginning of failure in clinical practice of the reconstruction of the ACL, meaning that the elongation is no longer elastic, but rather plastic. In other words, the deformity of the tested body is permanently irreversible 24,25.

Maximum tension is defined as the ratio between the maximum force of failure and the area of the graft. In the same way, the tension at the limit of proportionality is the ratio between the force at the limit of proportionality and the area of the graft. Rigidity, on the other hand, is considered as the capacity of a body to resist elongation by an applied force, and is defined as the ratio of maximum force and elongation.

Statistical analysis was performed with the aid of SPSS V17, Minitab 16, and Office Excel 2010. Due to the small sample size, we compared groups A and B using the Mann-Whitney test. To verify the degree of relation between the variables, Spearman's Correlation Test was used. We analyzed the groups both separately and together. The statistical significance level was established at 0.05 (type I error).

## RESULTS

All failures occurred for avulsion in the tibial fixation, and there were no incidences of graft rupture in its substance, or in its fixation in the hook of the machinery.

The results of the comparison of quantitative variables between groups A and B are shown in Table 1. Significant differences were found between the groups in the following variables: Maximum force (*p*=0.016), maximum tension (*p*=0.019), maximum deformation (*p*=0.007), and deformation at the limit of proportionality (*p*=0.003).

**Figure 6:**
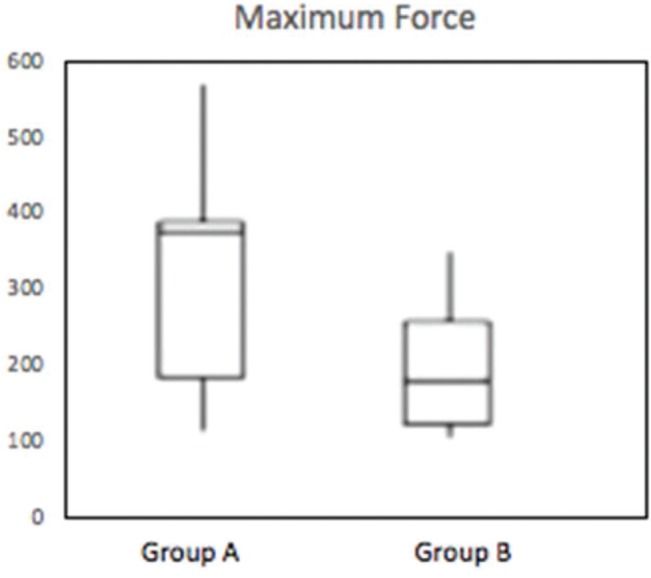
Box-plot demonstrating the distribution of the maximum force (N) of groups A and B (*p*=0.016).

**Figure 7:**
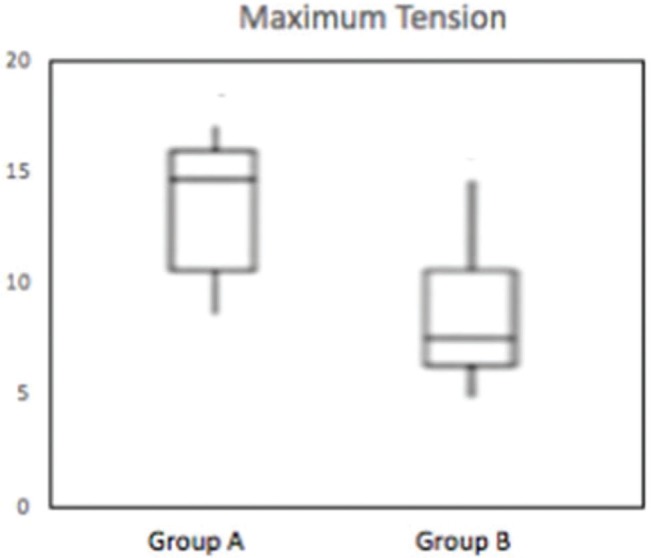
Box-plot demonstrating the distribution of maximum tension (N/mm^2^) of groups A and B (*p*=0.019).

**Figure 8:**
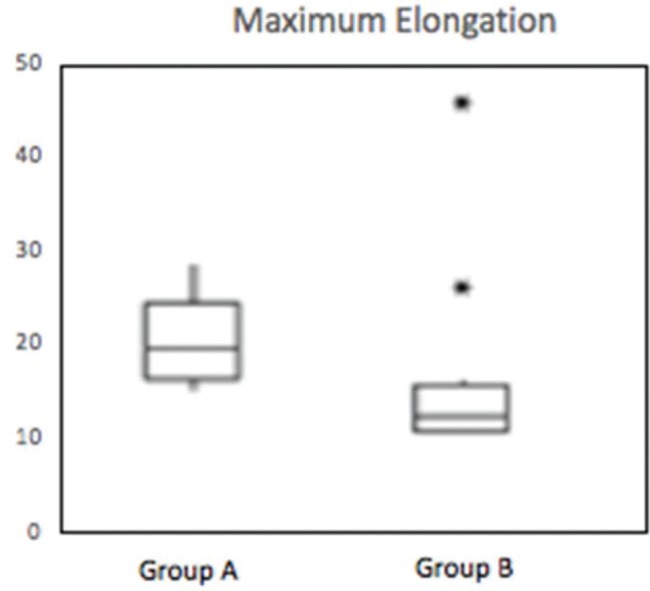
Box-plot demonstrating the distribution of maximum elongation (mm) of groups A and B (*p*=0.007). * Outlier.

**Figure 9:**
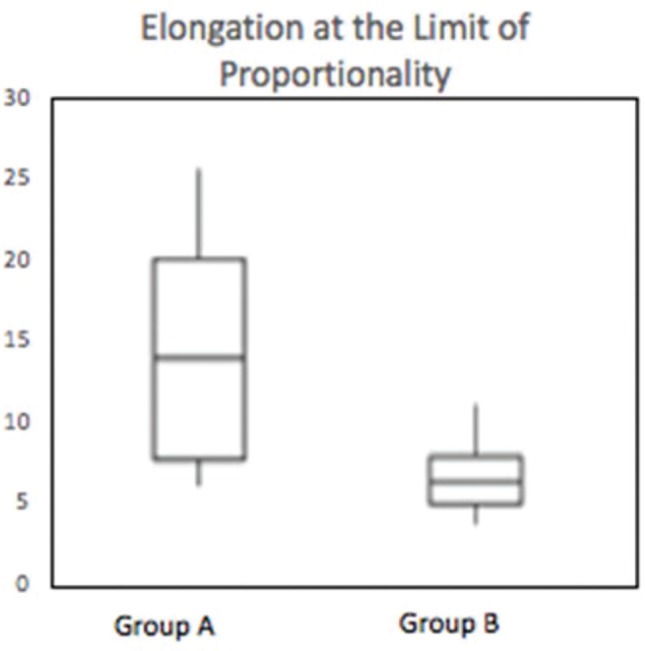
Box-plot demonstrating the distribution of the elongation at the limit of proportionality (mm) of groups A and B (*p*=0.003).

These differences can be observed in Figures 6, 7, 8 and 9.

Considering both groups together, we observed a moderate degree of negative correlation between area and rigidity (*p*=0.014; Corr=-0.568), and a strong positive correlation between MF and force at the limit of proportionality (*p*<0.001; Corr=0.764).

When correlating the variables considering solely group A, we found a strong positive correlation between area and maximum force (*p*=0.012; Corr=0.787), as well as between maximum force and force at the limit of proportionality (*p*=0.003; Corr=0.800).

Considering only group B, we observed a strong correlation between area and tension at the limit of proportionality (*p*=0.025; Corr=0.733)

## DISCUSSION

ACL double-bundle reconstruction preserving the native insertion of the hamstring tendons would provide economic benefits as it uses half the fixation devices of the usual technique 21.

We opted to use anatomical pieces of human knees *in vitro* hoping to approximate the results found to the possible clinical implications extrapolated to *in vivo* models 25-27.

We performed the trial using traction through the axis of the tunnel with a progressive increase in force and with a constant speed of deformation (20 mm/min). The trial machine measured the instantaneous load applied 4,13, and the trial was performed until final failure of the fixation, considered to be reached when the disconnection between the graft fastened to the hook and the tibia. In our study, similar to the observations of Kurosaka et al. 28 and Chen et al. 27, all failures occurred during tibial fixation; there were no cases of rupture of the graft in its substance or in its fixation to the machinery's hook.

Similar to previous findings, the traction force on the graft was applied in the same direction as the tunnel, adjusting the angulation of the tibia in the immobile support as necessary. Though this model does not represent the anterior shear force that is frequently involved in ACL rupture, it remains one of the most used models in this type of trial 17,25. We prefer this model to the application of force parallel to the long axis of the tibia, as we believe that the evaluation of the fixation is less influenced by other factors, such as angulation of the tunnel, graft abrasion at the exit curve, and an increase in resistance due to the winding path of the graft relative to the axis of application of the force.

The loads observed, force at the limit of proportionality and maximum force of failure, in the present study were smaller than those previously described; the latter usually has values around 350N–650 N for the fixation of flexor tendons in the tibia with interference screws 17,24,25. This difference can be explained by the different method of conservation of the knees, as frozen tendons have a smaller absolute value of elasticity than fresh tendons 26. Furthermore, it is known that fixation with interference screws is directly related to bone density, which is different for age and sex, as well as *in vivo* and *in vitro*^17^,^29^. As evidenced in another study using cadavers of patients who died at an advanced age, the maximum force was only 125 N, a value similar to our own results, which is likely related to the smaller bone density of elderly patients 29. Another factor that contributed to this discrepancy was the difference between human tissues and animal tissues of the various species that have been referenced in previous studies 25,26,28. It is also valid to highlight that the fixation evaluated in the previous studies is performed using a quadruple bundle of the semitendineous and gracilis, as opposed to the double bundle that was used in our study 17,24,25,29,30. Despite the discrepancies with previous studies involving the biomechanics of the tibial fixation of the flexor tendons with interference screws, we consider the current results to be valid, as the two groups were randomly divided from a greater homogeneous group, and a comparison between the two fixation techniques was possible. Therefore, tibial fixation, by preservation of the insertion of the hamstring tendons, was better in relation to the fixation obtained through interference screws, both for force at the limit of proportionality, which represent failure in clinical practice, and for maximum force 24,25. This, we can infer that tibial fixation through preservation of the tendon insertions as grafts in double bundles is superior to the fixation of this loose bundle with interference screws. Moreover, considering the possible low bone density present in our specimens, similarly to Nagarkatti et al. 29, the maximum force found in group A meets the initial fixation necessities of the graft at close to 350N, and is probably even greater in young patients with a higher bone density.

The preservation of the the insertion of the tendons of the gracilis and semitendineous muscles also has the theoretical advantage of speeding the vascularization of the graft, optimizing the ligamentization process, since the insertion can function as a vascular pedicle 7,31. Although this technique is currently used, it is not known precisely when the necrosis of these tendons occurs, with the consequential loss of the fixation and of the pedicular vascularization potential 32. However, the preservation of the insertion of the pes anserinus functions as an extra-tunnel fixation, and, in contrast to the intra-tunnel fixation provided by the interference screw, confers greater contact between the graft and the wall of the tunnel. As previously demonstrated, extra-tunnel tibial fixation promotes improved ligamentization by maintaining the force and rigidity of the graft-bone complex for longer than the intra-tunnel fixation (since the screw usually limits the total circumferential contact of the graft with the tunnel) 33.

This study has a number of limitations. First, the bone density of the cadaver piece was not measured and it was likely different to that found *in vivo*. Second, the age of death of the cadavers, as well as the sex (which could not be determined), also contributes to different mineral densities, which implies differences in the biomechanical quality of the grafts 34,35. Third, the traction exerted on the graft by the Kratos universal testing machine does not represent the same direction of restriction force to the anteriorization of the tibia that usually occurs in ACL lesions. Finally, the use of interference screws with a diameter two sizes bigger than the diameter of the graft, which is usual in clinical practice, was not tested, and the reconstructions were not submitted to cyclic loads.

## CONCLUSION

The use of the native insertion of the semitendineous and gracilis tendons, with no additional fixation device in the reconstruction of the postero-lateral bundle of the ACL showed mechanical superiority when compared to the fixation of these tendons with interference screws.

### Conflicts of Interest

Carlos Eduardo Franciozi is a consultant for Smith & Nephew.

## AUTHOR CONTRIBUTIONS

Santos AA was responsible for the design of the manuscript, data acquisition, analysis and interpretation, and manuscript writing and drafting review. Carneiro-Filho M was responsible for the study conception, acquisition of cadaver knees, and reviewing the draft of the manuscript. Albuquerque RFM was responsible for the acquisition of cadaver knees and availability of the biomechanical laboratory. De Moura JPFM was responsible for the acquisition, analysis, and interpretation of data. Franciozi CE was responsible for the design of the manuscript, the analysis and interpretation of data, and for writing and reviewing the draft of the manuscript. Luzo MVM was responsible for reviewing the draft of the manuscript. All authors approved the final version of the manuscript.

## Figures and Tables

**Table 1:** Comparison of the means according to the Mann-Whitney test.

	Mean ± standard deviation		
Variable	Group A	Group B	p*
Mean width of the graft (mm)	7.81±1.17	7.78±1.10	1.000
Mean area of the graft (mm^2^)	21.7±6.26	23.86±3.69	0.453
Maximum force (N)	315.4±124.7	195.7±71.8	0.016
Maximum tension (N/mm^2^)	13.57±3.65	8.80±3.81	0.019
Maximum elongation (mm)	19.73±4.76	15.30±10.73	0.007
Force at the limit of proportionality (N)	240.6±144.0	150.1±68.7	0.242
Deformation at the limit of proportionality (mm)	14.37±6.58	6.85±2.42	0.003
Tension at the limit of proportionality (N/mm^2^)	19.17±5.64	19.17±5.64	0.498
Rigidity (N/mm)	28.54±17.01	32.39±16.45	0.424

* Descriptive level obtain in the Mann-Whitney test for each of the variables compared.
